# The α-dystrobrevins play a key role in maintaining the structure and function of the extracellular matrix–significance for protein elimination failure arteriopathies

**DOI:** 10.1186/s40478-021-01274-8

**Published:** 2021-10-21

**Authors:** Matthew MacGregor Sharp, Jordan Cassidy, Thomas Thornton, James Lyles, Abby Keable, Maureen Gatherer, Masato Yasui, Yoichiro Abe, Shinsuke Shibata, Roy O. Weller, Dariusz C. Górecki, Roxana O. Carare

**Affiliations:** 1 Faculty of Medicine, University of Southampton, Southampton General Hospital, Tremona Road, Southampton, SO16 6YD UK; 2 Keio University School of Medicine, Tokyo, Japan; 3 Molecular Medicine, School of Pharmacy and Biomedical Sciences, University of Portsmouth, Portsmouth, England; 4 Military Institute of Hygiene and Epidemiology, Kozielska 4, 01-001 Warsaw, Poland

## Abstract

The extracellular matrix (ECM) of the cerebral vasculature provides a pathway for the flow of interstitial fluid (ISF) and solutes out of the brain by intramural periarterial drainage (IPAD). Failure of IPAD leads to protein elimination failure arteriopathies such as cerebral amyloid angiopathy (CAA). The ECM consists of a complex network of glycoproteins and proteoglycans that form distinct basement membranes (BM) around different vascular cell types. Astrocyte endfeet that are localised against the walls of blood vessels are tethered to these BMs by dystrophin associated protein complex (DPC). Alpha-dystrobrevin (α-DB) is a key dystrophin associated protein within perivascular astrocyte endfeet; its deficiency leads to a reduction in other dystrophin associated proteins, loss of AQP4 and altered ECM. In human dementia cohorts there is a positive correlation between dystrobrevin gene expression and CAA. In the present study, we test the hypotheses that (a) the positive correlation between dystrobrevin gene expression and CAA is associated with elevated expression of α-DB at glial-vascular endfeet and (b) a deficiency in α-DB results in changes to the ECM and failure of IPAD. We used human post-mortem brain tissue with different severities of CAA and transgenic α-DB deficient mice. In human post-mortem tissue we observed a significant increase in vascular α-DB with CAA (CAA vrs. Old p < 0.005, CAA vrs. Young p < 0.005). In the mouse model of α-DB deficiency, there was early modifications to vascular ECM (collagen IV and BM thickening) that translated into reduced IPAD efficiency. Our findings highlight the important role of α-DB in maintaining structure and function of ECM, particularly as a pathway for the flow of ISF and solutes out of the brain by IPAD.

## Introduction

Cerebral blood vessels serve a dual function; forming an essential component of the neuro-vascular unit to regulate cerebral blood flow [1] and providing extracellular matrix (ECM) pathways within the walls of capillaries, arterioles and arteries for the flow of cerebrospinal fluid (CSF) into the brain by convective influx/glymphatic flow [2, 3] and for the interstitial fluid (ISF) and solutes out of the brain by intramural periarterial drainage (IPAD) [4–8]. CSF tracer studies in rodents show that glymphatic flow occurs in the direction of blood flow, facilitated by the action of glial aquaporin 4 (AQP4), although the contribution of AQP4 is debated in the field [3, 9]. ISF and solutes flow out of the brain along the fused endothelial and glial basement membranes of capillaries in the parenchyma and continue towards the basement membranes surrounding smooth muscle cells in the arterioles and arteries, as IPAD [7, 10–12]. Failures in this process are linked to Protein Elimination Failure Arteriopathies, such as Cerebral Amyloid Angiopathy (CAA).

Pathological characteristics of CAA consist of the progressive accumulation of congophilic amyloid peptides of different amino acid lengths in the walls of leptomenigeal arteries, and in the walls of cortical arterioles and capillaries, very rarely affecting venules [13, 14]. Based on this pattern of amyloid deposition in both humans [13, 14] and transgenic mouse models, as well as experimental studies involving the study of drainage of tracers from the brain, it is recognised that a key mechanism for the development of CAA is the failure of clearance of amyloid along vascular basement membranes represented by IPAD [4, 15–19].

**Fig. 1:**
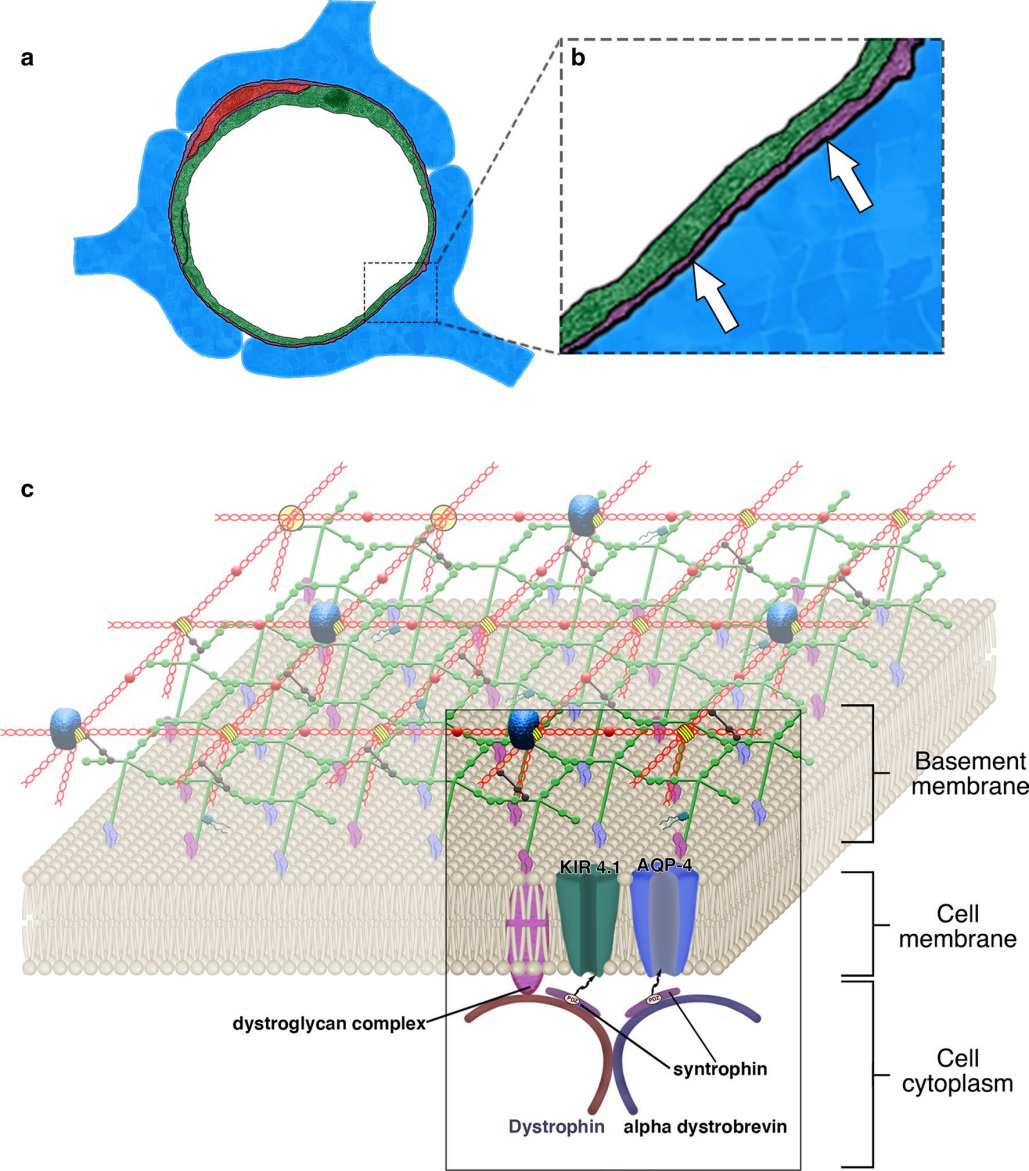
The dystrophin associated protein complex at astrocyte endfeet. **a** Astrocyte endfeet (blue) are anchored to the basement membrane (extracellular matrix) (purple) via the dystrophin associated protein complex located on the astrocyte cell membrane (**b**, white arrows). **c** The dystrophin associated protein complex. Transmembrane proteins are localised to the cell membrane by C-terminal tail binding to syntrophin. The dystroglycan complex anchors the cell membrane to laminin/agrin of the extracellular matrix. Dystrophin forms a bridge between the cell cytoskeleton and the cell membrane. The ECM in C is adapted from [32, 33]

The complex network of glycoproteins and proteoglycans of the ECM that constitute the IPAD pathway ensheath the abluminal side of endothelia, separating the endothelia from intramural cells (pericytes or smooth muscle cells) and the intramural cells from the endfeet of astrocytes, thus forming distinct basement membranes (BM) between the different cell types [20, 21]. Astrocyte endfeet are tethered to the BM by the interactions of laminin/agrin with integrin adhesion receptors and the dystroglycan complex, a transmembrane domain of the dystrophin associated protein complex (DPC). The dystroglycan complex is localised at the cell membrane by dystrophin and its associated dystrobrevin and syntrophin proteins [22]. Dystrophin is bound to intracellular cytoskeleton proteins such as actin, effectively forming a bridge between the cytosol and surrounding BM. Alpha-dystrobrevin (α-DB), the most prominent dystrobrevin isoform within perivascular astrocyte endfeet [23, 24], is an essential organiser of dystrophin-associated protein, showing continuous expression throughout development in BBB models. This is unlike dystrophin, highlighting the role of α-DB in maintaining BBB function [25]. α-DB shares homology to the cysteine-rich C-terminal region of dystrophin, binding with its reciprocal regions [26]. The syntrophins interact with α-DB and dystrophin to provide multiple anchor points for transmembrane proteins, important for the localisation of the potassium channel KIR4.1 and the water channel aquaporin 4 (AQP4) at the glial vascular interface [27, 28] (Fig. 1). This localisation is lost in mice deficient in α-DB, in a similar way to that observed in post-stroke dementia [25, 29, 30]. AQP4 has been implicated in glymphatic/convective influx of cerebrospinal fluid into the brain but its role in the tethering of astrocyte endfeet to the BM is unclear [31].

In α-DB deficient mice, astrocytes show reduced levels of syntrophin and dystrophin [34] and there is age-related modifications to the vessel wall, such as expanded ECM (BM) [25]. Remodelling of the ECM is a common feature of ageing and neurodegeneration, often associated with changes in expression of laminin and COL4 [35–39]. Although astrocytes cultured from α-DB deficient mice have been demonstrated to produce abnormal ECM with less laminin [25], the biochemical profile of the ECM and thus the effect of α-DB on COL4 has not been investigated in-vivo. This may be important in the context of protein elimination failure arteriopathies, as alterations to the ECM have been shown to impede the elimination of ISF by IPAD, favouring the accumulation of amyloid proteins as CAA [36, 40–43]. In a recent study using human cohorts, changes in astroglial gene products were observed to be associated with dementia status. In particular, there was a positive correlation between dystrobrevin gene expression and Protein Elimination Failure Arteriopathies [44], highlighting a potential role for the dystrobrevins and DPCs in regulating astrocyte-endfeet interactions with ECM in neurodegenerative disease. The role of the dystrobrevins and the DPC in the regulation of astrocyte-endfeet interactions and vascular ECM in heathy vasculature has received little attention and is relatively unknown. In the present study, we test the hypotheses that (a) the positive correlation between dystrobrevin gene expression and Protein Elimination failure Arteriopathies is due to elevated expression of α-DB at glial-vascular endfeet and (b) a deficiency in α-DB and loss of its function of binding AQP4 to glial-vascular endfeet results in changes to the ECM and IPAD pathways that promote Protein Elimination Failure Arteriopathies.

## Methods

### Animals

Mice deficient for AQP4 and C57BL/6 wild-type controls were generated by Ikeshima-Kataoka et el. at Keio University, Japan [45], who kindly donated fixed brains perfused and processed for TEM following our published protocols [4, 46]. Mice deficient for α-DB [29] were purchased from The Jackson laboratory (B6;129-Dtna tm1Jrs/J, 010,976) and bred in the Biomedical Research Unit, University of Southampton. All Mice were kept on a standard 12-h light/dark cycle and allowed food and water ad libitum. All procedures carried out at the University of Southampton were in accordance with animal care guidelines stipulated by the United Kingdom Animals (Scientific Procedures) Act 1986, Home Office licence P12102B2A.

### Human tissue samples

Sections of 10 μm thickness of post-mortem human occipital cortex were used in this study. Human tissue from young post-mortem donors (≤ 60 years old) was supplied by the MRC funded Edinburgh Sudden Death Tissue Brain Bank (Ethics REC 16/ES/0084). Tissue from old (≥ 65 years old) and CAA-affected post-mortem donors was supplied by the Newcastle Brain Tissue Resource (Ethics REC 08/H0906/136 + 5). The demographics of the cases used are summarised in Table 1. The cases from the MRC Sudden Death Brain & Tissue Bank (Edinburgh) had no neurological disease during life and no significant neuropathological changes post-mortem. As the cardiovascular and cerebrovascular risk factors are important in the pathogenesis of CAA, we have listed the available information in Table 1.

**Table 1:** Case demographics

Source	Age	Sex	pm delay/hr	Category	Risk factors
Edinburgh	50	M	46	Young	Hypercholesterolaemia
Edinburgh	50	M	84	Young	Hypercholesterolaemia
Edinburgh	49	M	79	Young	Asthma
Edinburgh	32	M	93	Young	Depression
Newcastle	79	F	9	old non-demented	Parkinson's disease
Newcastle	72	M	89	old non-demented	Angina
Newcastle	81	M	52	old non-demented	Hypertension
Newcastle	89	F	98	old non-demented	Congestive heart failure
Newcastle	75	M	82	CAA—dementia	Ischaemic heart disease, hypertension, type 2 diabetes
Newcastle	79	M	13	CAA—no dementia	Ischaemic heart disease, stroke, hypercholesterolaemia
Newcastle	87	F	54	CAA—dementia	Stroke
Newcastle	77	M	78	CAA—dementia	Hypertension

### Immunohistochemistry—human tissue

Two adjacent tissue sections of 10 µm thickness of the occipital grey matter from each case were deparaffinised and then rehydrated through a graded series of alcohols. Endogenous peroxidase activity was quenched with 10% hydrogen peroxide for 15 min at room temperature. Heat mediated antigen retrieval was then performed by microwaving in 0.01 M citrate buffer (pH 6) for 15 min. After blocking in 15% goat serum (Sigma, 9023) for 15 min at room temperature, sections were incubated in anti- α-DB primary antibody (1:25; Santa Cruz) or anti-collagen IV (1:100, Abcam) overnight at 4 °C in a moist chamber. Sections were then washed 3 × 10 min in 0.01 M phosphate buffered saline (PBS), incubated for 1 h in Biotinylated goat anti rabbit IgG (1:400, Vector) and then incubated at room temperature in ABC vector (Vector laboratories, PK-6100) for 1 h. Sections were developed using glucose oxidation enhancement with 3,3'-Diaminobenzidine (DAB) (Sigma-Aldrich, UK) for 5 min, dehydrated, cleared in xylene and coverslipped using DPX mounting medium (Sigma-Aldrich, UK).

An Olympus dotSlide digital virtual microscope was used to produce high powered tile scans of each section (magnification of × 40) (1 × section per case). Tile scans were imported into VS Desktop imaging software (Olympus) to produce 4 regions of interest (ROI)s 0.5 mm in width by 0.5 mm in height with an area of 1mm^2^ of occipital grey matter for further image analysis. Each ROI was imported into Adobe Photoshop CS6 and assessed for either vessel density, calculated by counting the number of COL4 positively vessels per 1 mm^2^, or for percentage of vessels stained for α-DB, determined by normalising the number of vessels stained for α-DB against vessel density and expressing as a percentage. Values generated for each ROI were combined to give total vessel density and total percentage of vessels positive for α-DB per section. Statistical analysis was performed using SPSS and an independent *t-test* with significance set at P < 0.05.

### Electron microscopy

10-week old α-DB deficient (n = 3), wild-type control mice (n = 3), AQP4 deficient (n = 2) and aged matched wild-type control mice (n = 2) were terminally anesthetised with pentobarbitone (200 mg/kg) and then intracardially perfused with 0.1 M piperazine-N,N’-bis (2-ethanesulfonic acid) buffer (PIPES, pH 7.2) followed by 4% formaldehyde plus 3% glutaraldehyde in 0.1 M PIPES buffer (pH 7.2). Brains were removed from the skull and subsequently placed in fresh 4% formaldehyde plus 3% glutaraldehyde in 0.1 M PIPES buffer (pH 7.2) at 4 °C for 24 h. Brains were microdissected for grey matter and processed according to our previous published protocols [46, 47]. Polymerised resin blocks were trimmed and sectioned using an Reichurt Ultracut E ultramicrotome (Reichurt, Germany). 80 nm ultrathin sections were cut, transferred onto copper grids (TAAB laboratories, UK) and counter-stained with lead citrate for 3 min. Grids were examined using a Tecnai T12 transmission electron microscope operating a Morada G3 digital camera and Radius image capture software (EMSIS, Münster, Germany). Each grid was methodically scanned from top right to bottom left. High resolution low power images of the first 20 capillaries in transverse section with well-defined and clearly resolved vessel walls from each mouse were digitally photographed. Each capillary was first analysed qualitatively for ultrastructural changes to capillary endothelium, BM and intramural cells. Capillaries were then analysed quantitatively for changes in expression of these components. Adobe Photoshop CS6 was used to manually demarcate the lumen, endothelium, intramural cells and BM, based on electron dense staining of lipid bilayers. Once demarcated, each feature was segmented, measured for surface area in µm^2^ and, other than the lumen, percentage area occupied of the vessel wall. Statistical analysis was performed using SPSS and a univariate Analysis of Variance (two-way Anova) adjusting for multiple sample points (40 per mouse) with significance set at P < 0.05.

### Immunohistochemistry-α-DB deficient mice

10-week-old α-DB deficient mice (n = 3) and wild-type control (n = 3) mice were terminally anesthetised with pentobarbitone (200 mg/kg) and then intracardially perfused with 0.01 M PBS followed by 4% Paraformaldehyde (PFA) in 0.01 M PBS, pH 7.4 at a rate of 5 ml min-1. Brains were dissected and post fixed for 6 h in fresh 4% PFA in 0.01 M PBS, pH 7.4 at 4 °C and then cryoprotected in 30% sucrose in distilled H_2_O at 4 °C for a further 48 h. Cryoprotected brains were removed from 30% sucrose and imbedded in OCT compound before serial sectioning into 20 μm coronal slices using a Leica CM1860 UV cryostat. Sections were collected onto SuperFrost Plus™ adhesion slides (Thermo Scientific™, 10,149,870) and stored at −20 °C.

Enzyme-linked immunohistochemistry using DAB (Sigma-Aldrich, UK) as chromogen was used to visualise COL IV positive vessels in the grey matter of α-DB deficient and wild-type control mice using 20 μm coronal frozen sections. One section per mouse was chosen based on matching the anatomy with sections used for TEM. Adjacent sections posterior and anterior to the chosen section were used as negative controls. After defrosting sections in an incubator at 37 °C for 15 min, endogenous peroxidase activity was quenched with 3% hydrogen peroxide (Sigma-Aldrich, UK) for 15 min at room temperature. Pepsin digest (1 mg/ml in 0.2 M hydrochloric acid) antigen retrieval was then performed at 37 °C for 4 min. After blocking in 15% normal goat serum (Sigma-Aldrich, UK) at room temperature for 30 min, sections were incubated with Rabbit anti-COL IV (1:400, abcam, ab6586) overnight at 4 °C in a moist chamber. This step was omitted with the negative controls to ascertain the level of background non-specific antigen binding of the tissue. Sections were then washed 3 × 10 min in 0.01 M PBS, incubated for 1 h in Biotinylated goat anti rabbit IgG (1:200, ThermoFisher Scientific, 31,820) and then incubated at room temperature in ABC vector (Vector laboratories, PK-6100) for 1 h. Sections were developed using glucose oxidation enhancement with DAB (Sigma-Aldrich, UK) for 12 min, dehydrated, cleared in xylene and coverslipped using DPX mounting medium (Sigma-Aldrich, UK).

An Olympus dotSlide digital virtual microscope was used to produce high powered tile scans of each section at a magnification of × 40 using 1 × section per mouse. Tile scans were imported into VS Desktop imaging software (Olympus) to produce 5 ROIs 250 µm in width by 250 µm in height with an area of 62,500 µm^2^ for further image analysis. Each ROI was assessed for DAB (COL4) staining using Image J [48] and a custom macro modified by colleagues from the Biomedical Imaging Unit, University of Southampton. Values generated for each ROI were combined to give a total number of vessels and total area of DAB staining per region per section. These values were then divided by the overall surface area (312,500 µm^2^) and multiplied by 500,000 and expressed as total number of vessels (vessel density) and total area stained per 0.5mm^2^. The amount of COL IV per vessel was calculated by dividing the total area stained by the total number of vessels. Each ROI was further assessed for differences in vessel distribution by counting the number of capillaries (vessels with diameter of less than 10 µm) and arterioles and venules which, which due to limitations of DAB staining, could not be easily differentiated and so were classified together as vessel with a diameter of 10 µm or larger. Inbuilt measurement and analysis tools in Adobe Photoshop CS6 were used to identify and count the number of vessels with a diameter of 10 µm or larger per ROI using imported layer masks of each ROI (generated from the ImageJ macros). The number of capillaries of each ROI was calculated by deducting the counted number of arterioles/venules from the overall total vessel count (generated previously using the ImageJ macros). Values generated for each ROI were combined to give the total number of capillaries and arterioles/venules per region per section. These values were then divided by the overall surface area (312,500 µm^2^) and multiplied by 500,000 and expressed per 0.5mm^2^. Adobe Photoshop CS6 was also used to measure and calculate overall COL IV staining per vessel type. First, the arterioles/venules identified previously from the layer masks of each ROI were measured for total area of COL IV staining. This value was then deducted from the overall area of COL IV staining to give total area of COL IV staining for capillaries per ROI. Values generated for each ROI were combined to give the total area of COL IV staining per capillaries and per arterioles/venules per region per section. These values were then divided by the overall surface area (312,500 µm^2^) and multiplied by 500,000 to be expressed per 0.5mm^2^. The amount of COL IV per vessel was calculated by dividing the total area stained by the total number of vessels for each vessel group per 0.5mm^2^. Statistical analysis was performed using SPSS and an independent *t-test* with significance set at P < 0.05.

### Assessment of IPAD in α-DB deficient mice

10-week-old α-DB deficient (n = 5) and wild-type control mice (n = 5) were anaesthetised with Isoflurane mixed with concentrated O_2_ (1.7 L min-1), induced with 3% and maintained using 2%. The level of anaesthesia was monitored by using pedal withdrawal reflex response. Internal body temperature was regulated at 37 °C using a rectal probe and homoeothermic blanket and temperature control system (BASi). Lacri-lube ointment was applied to the eyes to preserve cornea during anaesthesia.

Anaesthetised mice were placed in a stereotaxic frame (Model 900, KOPH instruments) and the head secured with jaw bars. After performing a midline incision, a Tech2000 Micromotor drill (RAM Products, INC) with 0.7 mm burr was used to create a burr hole in the skull above the injection site (Anterior–Posterior—2 mm; Medial–Lateral 1.5 mm; Dorsal–Ventral—1.7 mm). 0.5 µl of 100 µM amyloid-β (1–40) HiLyte Fluor 555 (Cambridge Bioscience) was injected into the hippocampus using a Hamilton Neuros Syringe with a 33-gauge needle (Essex Scientific Laboratory Supplies Ltd.) and Microinjection syringe pump (UMP3T-1; World Precision Instruments) at a rate of 0.25 µl min-1. The hippocampus was chosen based on the occurrence of CAA in the hippocampus of Amyloid precursor protein (APP) transgenic mouse models [49, 50]. The syringe was left in situ for 2 min for bolus diffusion and to prevent reflux. The tracers were left to drain for a further 5 min and then the mouse was terminally anaesthetised with pentobarbitone (200 mg/kg) and intracardially perfused with 0.01 M PBS followed by 4% PFA in 0.01 M PBS, pH 7.4 at a rate of 5 ml/min. Brains were dissected and post fixed for 6 h in fresh 4% PFA in 0.01 M PBS, pH 7.4 at 4 °C and then cryoprotected in 30% sucrose in distilled H_2_O at 4 °C for a further 48 h. Brains were embedded in OCT compound and then sectioned into 20 μm coronal slices using a Leica CM1860 UV cryostat. Sections were collected onto SuperFrost Plus™ adhesion slides (Thermo Scientific™, 10,149,870) and viewed using a Zeiss Axioskop 2 fitted with a rhodamine filter to identify the section containing the site of injection. Our previous studies showed that the drainage of Aβ40 in mice occurs predominantly in a posterior direction and can be visualised in the walls of blood vessels as close as 200 μm to the injection site [51]. We therefore chose coronal Sects. 200 μm posterior to the injection site for immunohistochemistry.

Sections were washed 2 × 3 min in 0.01 M PBS, pH 7.4 and then blocked in 15% goat serum (Sigma 9023) for 1 h at room temperature. Sections were then incubated in rabbit anti-collagen IV 1/400 in 0.01 M PBSt (AbCam, ab6586) and anti-smooth muscle actin (SMA) FITC conjugated 1/200 in 0.01 M PBSt (Sigma, F3777) overnight in a moist chamber at 4 °C. Sections were then washed 3 × 10 min in 0.01 M PBS and incubated in conjugated secondary antibody goat antirabbit Alexa Fluor 633 0.01 M PBSt (ThermoFisher Scientific, A-21070) for 1 h at room temperature. Sections were further incubated in 1% Sudan Black for 5 min to remove auto fluorescence before being mounted in Mowiol Citifluor and imaged using confocal microscopy.

For each section, tile scans of the left hippocampus or corpus callosum were captured using a Leica SP8 confocal microscope fitted with × 20 objective set at an optical zoom of 1. Laser power and detection windows were kept consistent for all scans. Sequential imaging was used to prevent cross excitation of fluorophores. Quantification of IPAD of Aβ40 HiLyte Fluor 555 was performed using a maximum projection of each tile scan uploaded into Adobe Photoshop CS6. IPAD was assessed by using Adobe Photoshop CS6 to manually measure vessel density of capillaries, arterioles and venules and counting the number of vessels containing Aβ40 HiLyte Fluor 555 in their vessel walls. Vessel density was calculated by dividing the total number of capillaries, arterioles or venules by the overall surface area (in µm^2^) and multiplying this value by 500,000 to be expressed as number of vessels per 0.5mm^2^. This was also performed for the number of capillaries, arterioles or venules with Aβ40 in their vessel walls. Vessels were identified based on lumen diameter and immunoreactivity to SMA (< 10 μm = capillaries, ≥ 10 μm and SMA positive = arterioles, ≥ 10 μm and SMA negative = venules [7, 51]. The overall surface area was determined by choosing regions of interest that were based on key anatomical features that could be observed in each section analysed. Regions of interest were outlined using Adobe Photoshop CS6. For the hippocampus, a transverse area from the edge of the suprapyramidal [52] was outlined. Statistical analysis was performed using SPSS Statistics version 26.0 (IBM) and an independent t-test with significance set at P < 0.05.

## Results

### Expression of α-DB at glial-vascular endfeet is elevated in protein elimination failure arteriopathies

**Fig. 2:**
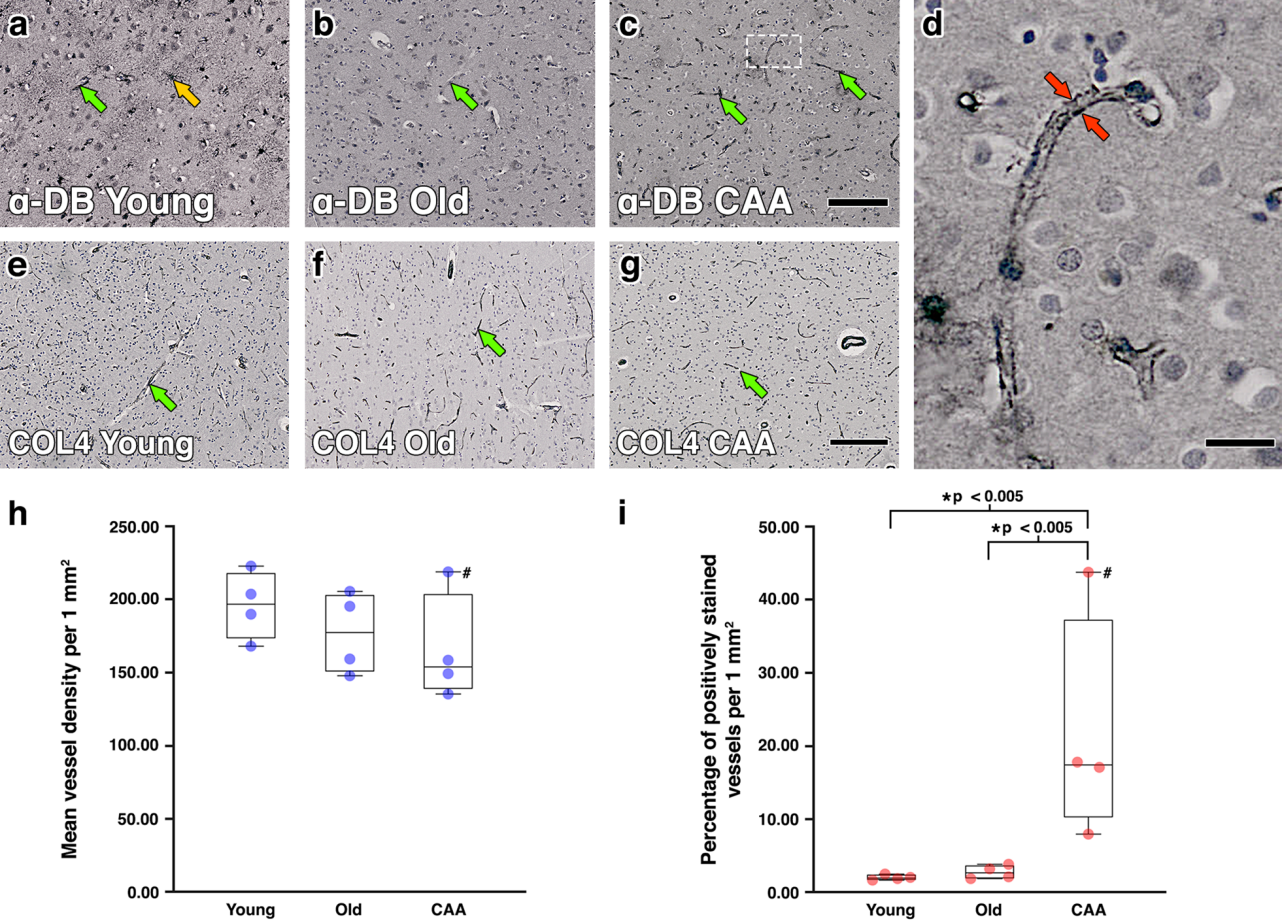
Expression of α-dystrobrevin (α-DB) in the grey matter of occipital lobe in young, old and CAA brains. Immunoreactivity for α-DB appeared greatest around cells with morphology similar to astrocytes in young cases (**a**, orange arrows) but also outlined blood vessels in all cases (**b, c**, green arrows), most notably in CAA cases with punctate staining consistent with astrocyte endfeet (**d**, red arrows). Scale bars **a**–**c** = 100 µm, **g** = 20 µm. Immunohistochemistry for COL4 revealed no notable differences in vessel staining (**e–g**, green arrows) or vessel density (**h**) between young, old or CAA cases. Scale bars **e–g** = 100 µm. When normalised against vessel density, the percentage of vessels positively stained for α-DB was significantly higher in CAA cases when compared to both old and young cases (**i**). Each data point in **h** and **i** represents overall mean values (n = 4) from each case. Data points labelled with **#** were identified as outliers and excluded from analysis

To assess whether the positive correlation between dystrobrevin gene expression and protein elimination failure arteriopathies in human dementia cohorts previously reported in [44] could be associated with elevated expression of α-DB at glial-vascular endfeet, post-mortem occipital brain sections from young, old non-demented and severe CAA brains were immunostained for α-DB. In all cases, astrocytes and blood vessels were found to be positively stained for α-DB, but the intensity of staining depended on age and disease. In tissue from young cases, immunoreactivity for α-DB appeared to be higher around cells with morphology similar to astrocytes while in severe CAA, immunoreactivity appeared greatest around blood vessels with punctate staining consistent with astrocyte endfeet (Fig. 2 a-d). Assessment of vessel density, shown by staining vessels for COL4, revealed a slight but non-significant reduction between young (196.5 vessels per 1mm^2^), old non demented (177.44 vessels per 1mm^2^) and severe CAA cases (165.88 vessels per 1mm^2^) (Fig. 2 e–h). Quantification of the number of positively stained vessels for α-DB, after normalisation against vessel density, revealed no significant differences between young (2.08%) or old (2.82%) cases. However, the percentage of vessels positive for α-DB in CAA cases (21.71%) was significantly greater compared to both old (p < 0.005) and young (p < 0.005) cases (Fig. 2 i).

### A lack of α-dystrobrevin alters the structure of IPAD pathways by altering ECM morphology

**Fig. 3:**
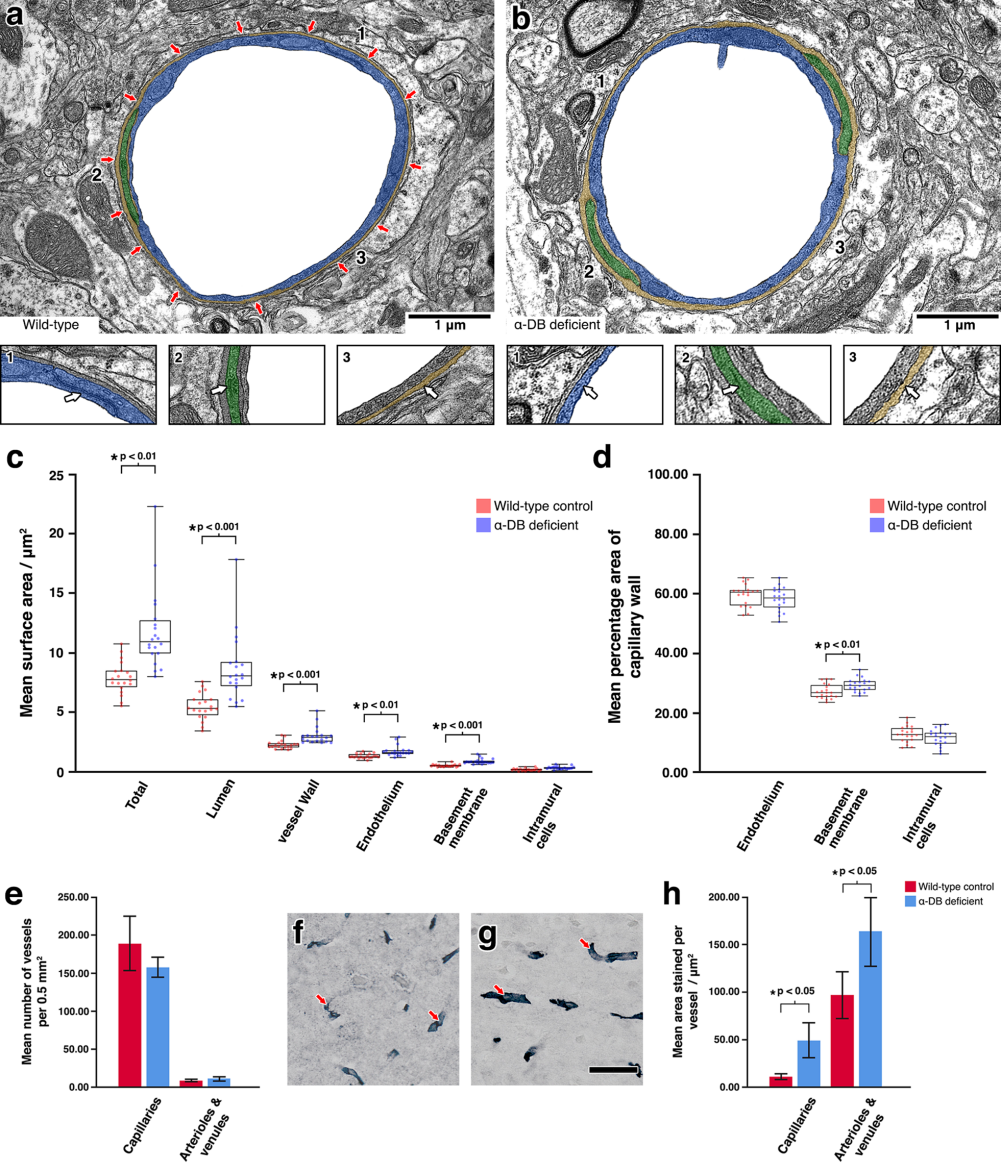
Analysis of the perivascular compartment in α-DB deficient mice. **a** and **b** Assessment by transmission electron microscopy revealed a thicker appearing basement membrane (false coloured orange) in α-DB deficient mice when compared to wild-type control mice (**a3** and **b3**). No other notable differences were observed between endothelium (**a1** and **b1**, false coloured blue) or intramural cells (**a2** and **b2**, false coloured green). The red arrows in (**a**) indicate the location of glial α-DB in wild-type control mice. When analysed for mean surface area, capillaries in α-DB deficient mice showed larger lumens and thicker vessel walls (**c**). The ratio of endothelium, intramural cells and basement membrane were altered with basement membrane occupying a significantly larger percentage area of the vessel wall (**d**). Each box plot represents the range of data from three mice. The scatter plots represent the means of repeated measures (20 per mouse, n = 3). Immunohistochemistry for COL4 revealed no notable differences in vessel density between α-DB deficient and wild-type control mice (**e**) but did show vessels with more intense staining (red arrows in **f** and **g**, scale bar 50 μm) in α-DB deficient mice. Expression of COL4 per vessel was found to be significantly higher in all vessel types in α-DB deficient mice (**h**). Each column represents overall mean values (n = 3). Error bars: ± 2 SE

In CAA, vessels laden with amyloid show severe ultrastructural abnormalities and alterations to the endothelium [53, 54], smooth muscle cells [54, 55] and intramural cells (pericytes) [56]. In severe CAA, extensive deposition of amyloid results in focal fragmentation of the vessel wall [56]. As vascular expression of α-DB significantly increased in cases of severe CAA, we next used quantitative electron microscopy on grey matter capillaries from α-DB deficient and aged matched wild-type control mice to investigate whether α-DB plays a key role in maintaining the structure of IPAD pathways. When compared to wild-type control mice, the structural appearance of grey matter capillaries in α-DB deficient mice appeared altered with a thickened BM. There were no notable differences in endothelium, intramural cells, astrocyte endfeet or adjacent parenchyma (Fig. 3 a and b). Quantitative assessment of capillaries by image segmentation revealed that capillaries in α-DB deficient mice were significantly larger in overall surface area (11.82 μm^2^ vs. 8.02 μm^2^, p < 0.01) with larger lumina (8.74 μm^2^ vs. 5.62 μm^2^, p < 0.001) and thickness of vessel wall (3.08 μm^2^ vs. 2.40 μm^2^, p < 0.001). This increase in vessel wall thickness was due to a thicker endothelium (1.79 μm^2^ vs. 1.43 μm^2^, p < 0.01) and BM (0.92 μm^2^ vs. 0.65 μm^2^, p < 0.001) (Fig. 3 c). To account for these differences in vessel size, the surface areas of the endothelium, intramural cells and BM were normalised against the overall vessel wall surface area and expressed as percentage occupied of the vessel wall. This showed that the surface area of the vessel wall occupied by BM was significantly increased in α-DB deficient mice (27.28% vs. 30.22%, p < 0.01). There was no difference between endothelium (58.78% vs. 59.82%, p = 0.490) or intramural cells compared to control wild-type mice (12.88% vs. 11.00%, p = 0.094) (Fig. 3d).

### Loss of α-dystrobrevin mediated localisation of AQP4 to glial vascular endfeet results in larger vessels but does not alter vessel wall morphology

**Fig. 4:**
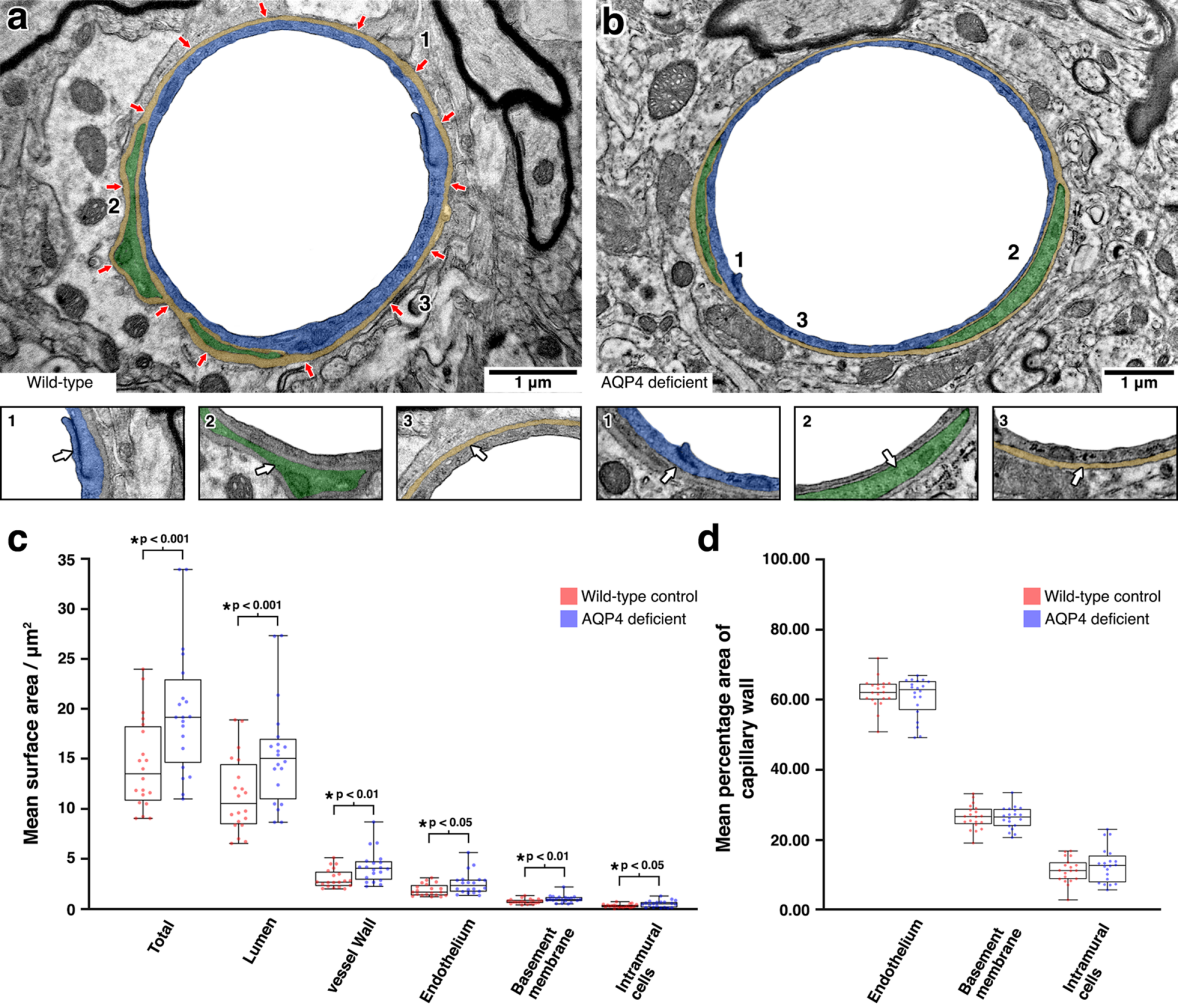
Analysis of the perivascular compartment in AQP4 deficient mice. **a** and **b** Assessment by transmission electron microscopy revealed no notable differences in morphology of the endothelium (**a1** and **b1**, false coloured blue), intramural cells (**a2** and **b2**, false coloured green) or basement membrane (**a3** and **b3**, false coloured orange) between wild-type control and AQP4 deficient mice. The red arrows in (**a**) indicate the location of glial AQP4 in wild-type control mice. When analysed for mean surface area, capillaries in AQP4 deficient mice showed larger lumens and thicker vessel walls (**C**). The ratio of endothelium/intramural cells or basement membrane were not significantly altered (**d**). Each box plot represents the range of data from two mice. The scatter plots represent the means of repeated measures (20 per mouse, n = 2)

As localisation of AQP4 to astrocyte endfeet is lost in mice deficient in α-DB [25], we next sought to ascertain if this could explain our observations of a thickened BM in α-DB deficient mice. We therefore repeated the ultrastructural study of the cerebral vasculature using mice deficient for AQP4. We found that when compared to wild-type control mice, the structural appearance of grey matter capillaries in AQP4 deficient mice appeared unaltered. There were no notable differences in endothelium, intramural cells, BM, astrocyte end-feet or adjacent parenchyma (Fig. 4 a and b). Quantitative assessment of capillaries by image segmentation revealed that capillaries in AQP4 deficient mice were significantly larger in overall surface area (19.90 μm^2^ vs. 14.53 μm^2^, p < 0.001) with larger lumina (15.58 μm^2^ vs. 11.45 μm^2^, p < 0.001) and thickness of vessel wall (4.32 μm^2^ vs. 3.08 μm^2^, p =  < 0.01). The endothelium (2.65 μm^2^ vs. 1.93 μm^2^, p < 0.05), BM (1.08 μm^2^ vs. 0.80 μm^2^, p < 0.01) and intramural cells (0.59 μm^2^ vs. 0.35 μm^2^, p < 0.05) were all significantly larger in AQP4 deficient mice (Fig. 4 c). As was the case with α-DB deficient mice, we next normalised this data against the overall vessel wall surface area. This revealed no significant differences in the surface area of the vessel wall occupied by the endothelium (60.98% vs. 62.13%, p = 0.530), BM (26.28% vs. 26.57%, p = 0.811) or intramural cells (12.83% vs. 11.30%, p = 0.298) (Fig. 4d), suggesting that our observations in α-DB deficient mice were not due to the loss of AQP4.

### A lack of α-DB is associated with biochemical changes to the ECM

Morphological changes to cerebral vascular BM, such as thickening, are associated with alterations to their biochemical composition. In particular, BM thickening has been linked to elevated levels of COL4 in both aging [37, 38] and in protein elimination failure arteriopathies, such as CAA [35, 57]. Previous studies by Lien et al. and our group show BM thickening in aged α-DB deficient mice but the biochemical composition of the BM was not assessed [25]. Therefore, we investigated whether alterations to the BM in α-DB deficient mice could be attributed to changes in expression of COL4, one of the main constituents of vascular BMs. Qualitative assessment by light microscopy was accompanied by quantitative analysis of the expression of COL4 by comparing the number of vessels (vessel density) and mean area stained per vessel. Qualitative assessment revealed that the majority of COL4 immunoreactivity was confined to vascular walls that appeared more intense and less diffuse in the α-DB deficient mice (Fig. 3 f and g). Assessment of vessel density revealed no significant difference in the distribution of COL4 stained capillaries between wild-type control (188 per 0.5 mm^2^) and α-DB deficient mice (156 per 0.5 mm^2^) p = 0.173. There was also no difference in the distribution of COL4 stained arterioles and venules (8 per 0.5 mm^2^ vs. 10 per 0.5 mm^2^, p = 0.275) (Fig. 3 e). However, in α-DB deficient mice, capillaries and arterioles and venules showed a significantly higher mean area staining of COL4 per vessel (capillaries, 49.25 μm^2^ vs 11.00 μm^2^, p < 0.05, arterioles and venules, 162.75 μm^2^ vs 96.27 μm^2^, p < 0.05) (Fig. 3 h). This suggests that basement membrane thickening in α-DB deficient mice is linked to alterations in COL4 expression, a finding not dissimilar to that observed with human ageing and dementia [35, 37, 38, 57].

### Biochemical changes to the basement membrane in α-DB deficient mice are linked to impaired Aβ clearance by IPAD

**Fig. 5:**
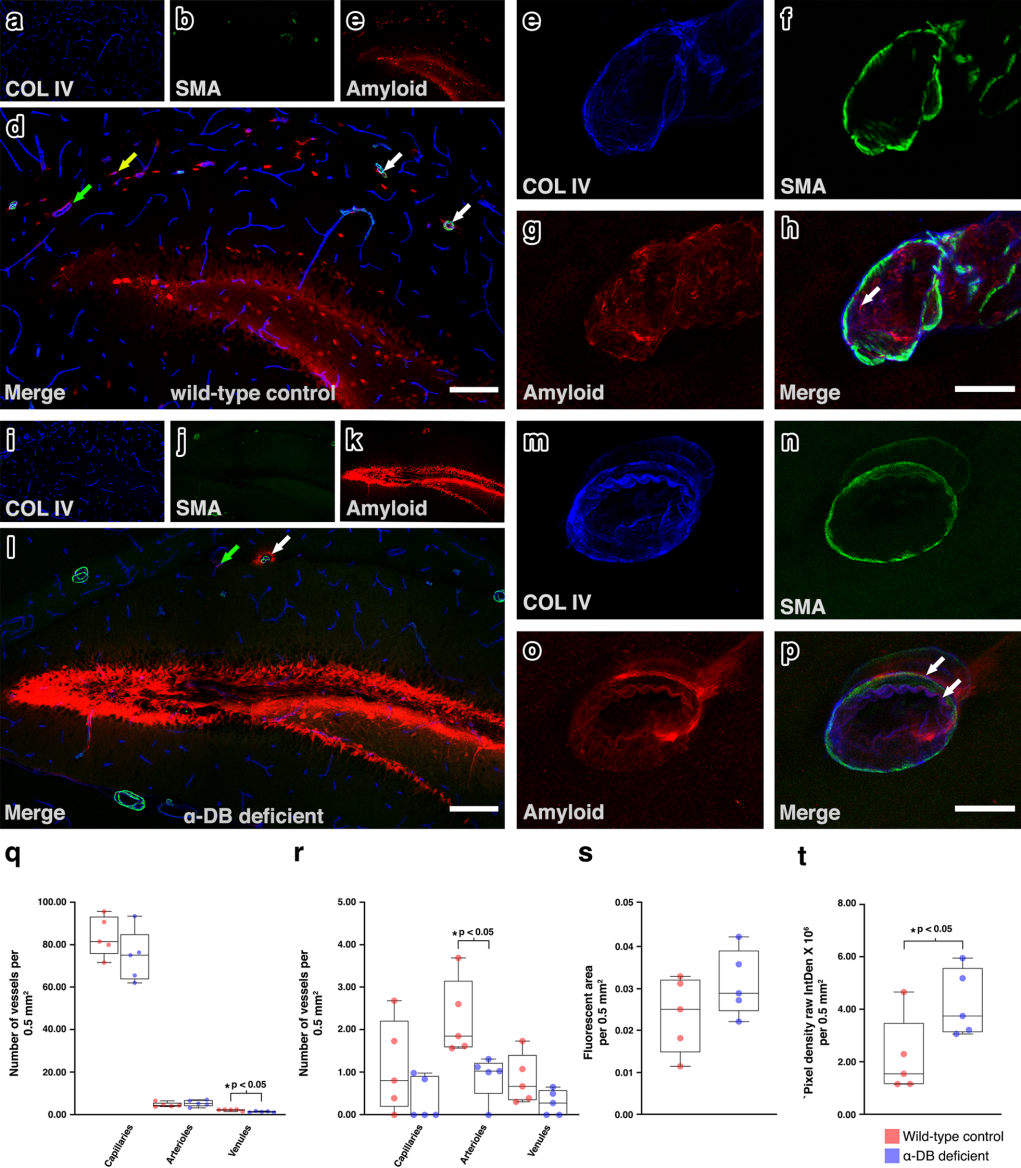
IPAD in α-DB deficient mice. In wild-type control mice, Aβ was observed diffusely distributed in the parenchyma (**e** and **d**) and co-localised with collagen IV in the walls of arterioles (white arrows), capillaries (yellow arrow) and few venules (green arrow). In α-DB deficient mice, the fluorescence due to Aβ appeared more intense in the parenchyma (**k** and **I**) but also co-localised with collagen IV in the walls of arterioles (white arrows) and few venules (green arrow). Representative high-power images of an arteriole shows amyloid-β (red) in the wall of the blood vessel, indicated by the white arrow in both wild-type control (**e**–**h**) and -DB deficient mice (**m**–**p**). Scale bars **a–d** and **I–l** = 200 µm, **e**–**h** and **m**-**p** = 10 µm. There was no difference in vessel density of capillaries or arterioles between wild-type control and α-DB deficient mice, but α-DB deficient mice showed both a significantly lower density of venules (**q**) and a significantly lower density of Aβ positive arterioles (**r**). The spread of Aβ in the parenchyma as measured by surface area was similar between wild-type control and α-DB deficient mice (**s**) but fluorescent intensity was significantly greater in α-DB deficient mice (**t**). Each box plot represents the range of data from five mice. The scatter plots represent vessel density (**q** and **r**), fluorescent area (**s**) or pixel density (**t**) from each mouse

The development of sporadic CAA is due to a failure of clearance of Aβ by IPAD [4, 15, 16]. As we observed an increase in vascular expression of α-DB in cases of CAA and alterations to vascular BMs in α-DB deficient mice, similar to that observed in the early stages of CAA [35, 57], we next investigated if removal of Aβ by IPAD would also be affected in α-DB deficient mice. To assess IPAD, α-DB deficient and wild-type control mice received a stereotaxic injection of Aβ HiLyte Fluor 555 into the hippocampus. The elimination Aβ by IPAD was assessed using confocal microscopy. Within 5 min of injection, fluorescent Aβ was observed in the granule cell layer of the parenchyma and colocalising with COL IV within the walls of arterioles, capillaries and few venules in both wild-type control (Fig. 5 a–h) and α-DB deficient mice (Fig. 5 i–p). In wild-type control mice, fluorescent Aβ was distributed diffusely (Fig. 5 d) while in α-DB deficient mice, Aβ appeared less diffuse and more focally concentrated within the parenchyma. Aβ was also seen aggregating around arterioles that did not have Aβ in their BMs, in a location consistent with glia limitans, a feature not observed in wild-type mice (Fig. 5 l). Assessment of the density of vessels with fluorescent Aβ in their vessel walls showed a significant reduction in Aβ positive arterioles in α-DB deficient mice compared to wild-type controls (2.27 vs 0.90 per 0.5 mm^2^, p < 0.05). The density of Aβ positive capillaries (1.13 vs 0.37 per 0.5 mm^2^, p = 0.193) and venules (0.84 vs 0.29 per 0.5 mm^2^, p = 0.98) also decreased in α-DB deficient mice but did not reach significance (Fig. 5 r). To confirm that these findings were not due to general differences in vessel density, we next assessed the same regions of interest for the vascular density of all arterioles, capillaries and venules, using immunoreactivity for COL4 as a vessel marker. There was no difference in the vessel density of arterioles (4.4 vs. 5.4 per 0.5 mm^2^, p = 0.270) or capillaries (84 vs. 74.7 per 0.5 mm^2^, p = 0.270) but the density of venules was significantly decreased in α-DB deficient mice (1.9 vs. 1.3 per 0.5 mm^2^, p < 0.05) (Fig. 5 q) suggesting that the decrease in amyloid positive arterioles and capillaries observed in α-DB deficient mice was not due to differences in vascular density. To further establish if the removal of Aβ by IPAD is impaired in α-DB deficient mice, we assessed the distribution of parenchymal Aβ by fluorescent density analysis. In α-DB deficient mice, there was an increase in mean area of fluorescent signal (0.02 mm^2^ vs. 0.03 mm^2^, p = 0.199) (Fig. 5 s) and a significant increase in mean fluorescent pixel density (2.18 × 10^6^ vs. 4.25 × 10^6^ pixels, p < 0.05) (Fig. 5 t), mirroring the qualitative observations of a more intense amyloid positive signal in α-DB deficient mice (Fig. 5 K and i). This suggests an increase in accumulation of Aβ in the parenchyma of α-DB deficient mice, further supporting the notion of impaired IPAD.

## Discussion

We used immunohistochemistry (IHC) for α-DB on grey matter from human post mortem samples to show a significant increase in the expression of α-DB in CAA compared to age-matched non-demented controls and young brains. To help understand why this might occur in neurodegenerative diseases affecting the vasculature, we next examined more closely the structural and functional consequences of a lack of α-DB on cerebral blood vessels. Previous studies in old α-DB deficient mice demonstrated abnormalities of the vascular walls and fluid homeostasis, leading to swollen astrocyte endfeet and tissue oedema [25]. Even though abnormalities in BBB function have been documented in α-DB deficient mice [25] other possible effects of α-DB deficiency on vascular ECM are not clear. To ascertain more clearly the exact consequence of α-DB deficiency on the cerebral vasculature, we performed a detailed quantitative ultrastructural study on the vessel wall using TEM, first in α-DB deficient mice and then in AQP4 deficient mice. We show that while a redistribution of AQP4 away from astrocyte endfeet does not appear to impact on the structure of the vascular wall, α-DB appears essential for vessel wall morphology. In α-DB deficient mice, we observed an increase in expression of COL4, a feature also concurrent to the early stages of CAA [35, 57]. Due to this similarity, we assessed the functional consequences of α-DB deficiency on the elimination of amyloid proteins by IPAD. We show that α-DB deficiency led to reduced efficiency of IPAD and the accumulation of amyloid in the parenchyma, highlighting a key role for α-DB and the DPC not only in the structure of the ECM but also in IPAD.

The lack of consistent morphological modifications to the vessel wall in our AQP4 deficient mice suggests it is not critical for the morphology of the vessel wall. However, the function of AQP4 in the regulation of cerebral fluid homeostasis has been well documented [3, 10, 58–60] and it is accepted that AQP4 plays a significant role in the formation or prevention of oedema [27, 61–67]. It has been suggested that a loss of both KIR4.1 and AQP4 from the glial-vascular interface would impair ion and water homeostasis and lead to the osmotic opening of tight junctions in response to changes in cell volume [25]. Ordinarily, a loss of glial AQP4 would impair the removal of excess water from the glial vascular interface [25, 68–70] without altering blood–brain barrier (BBB) function [62, 63, 67, 71]. However, in α-DB deficient mice, BBB function is impaired due to an increase in permeability of the endothelia [25]. Examination of the cerebral vasculature in 20-month-old α-DB deficient mice using transmission electron microscopy (TEM) reveals this is most likely due to damaged endothelium and abnormal tight junctions, highlighting the importance of α-DB and the DPC to BBB structure and function [25]. We observed no evidence of vasogenic oedema in young α-DB deficient mice or AQP4 deficient mice. However, our assessment of the cerebral vasculature in young α-DB deficient mice revealed similar changes to the BM as previously observed by Lien et al. [25], although we did not observe any modifications to the endothelium or astrocyte endfeet, most likely due to the younger age of our mice. In our α-DB deficient mice we showed thicker BM that occupied a larger area of the vessel wall, similar to aged wild-type control mice [35], suggesting that the cerebrovasculature in α-DB deficient mice show signs of premature ageing. Examination of the cerebral BM in more detail revealed an increase in expression of COL4, a feature frequently observed in aged human brains and CAA [37, 38]. Considering that α-DB is entirely an intracellular protein, its role in the formation and maintenance of the BM is likely due to indirect interactions via the DPC. Its deletion from the glial vascular interface may affect other interactions between DPC components, such as a weakening of the connection between α-dystroglycan and BM. This may stimulate a compensatory over production of ECM resulting in a thickened BM with altered morphology.

Regulation of the ECM is in part, due to the activity of matrix metalloproteinases (MMP)s and tissue inhibitors of metalloproteases (TIMP)s [72]. In particular, the matrix metalloproteinase MMP9, also known as Gelatinase B, is a COL4 collagenase that also digests β-dystroglycan in response to enhanced synaptic activity [73]. α-DB deficient astrocytes show reduced levels of β-dystroglycan [25] and although not confirmed in α-DB deficient mice, the loss of β-dystroglycan as an MMP9 substrate may disrupt the regulation of the ECM by MMP9, leading to the alterations to the BM. It is important to note that while changes to COL4 are a key contributor to BM remodelling, other BM proteins, such as the laminins can also play a significant role [35, 57]. Even though we only analysed COL4 in our α-DB deficient mice, a lack of α-DB is associated with abnormal levels of laminin [25]. It is therefore likely that the BM remodelling in our α-DB deficient mice is in part, also due to alterations in laminin and other BM proteins, highlighting the need to clarify the exact BM components that are at risk from the modifications of the DPC.

The failure of clearance of amyloid along IPAD pathways is a key pathogenic factor in CAA [4, 15, 16]. Mathematical modelling by our group has highlighted the importance of the BM to the process of vasomotion, a force generated by cycles of contraction and relaxation of smooth muscle cells that induces BM deformations, effectively opening and closing a valve like system allowing the flow of IPAD in the direction of the vasomotor wave [74]. Vasomotion is sensitive to alterations to the biochemical composition of the BM. Elevated levels of COL4, as we observed in our α-DB deficient mice would most likely stiffen the vessel wall, reducing deformations induced by the SMCs and altering IPAD. Our α-DB deficient mice demonstrated a reduced capacity of the IPAD pathway, highlighting the importance of α-DB and the DPC to the clearance of fluid from the brain via IPAD. One limitation of the study is that all the CAA cases used, all but one of the non-demented control brains and two out of the four young brains had cerebrovascular risk factors prior to death and it is not known what impact these risk factors had on the current analysis.

## Conclusion

Our work has demonstrated that α-DB is key in maintaining the morphology and composition of cerebral BMs and a normal IPAD, with a significant increase in α-DB in human CAA.

## Data Availability

The datasets used and/or analysed during the current study are available from the corresponding author on reasonable request.
